# Baseline β-Cell Secretory Reserve and Its Association with Glycaemic Control and Long-Term Outcomes Across Diabetes Phenotypes

**DOI:** 10.3390/ijms27042035

**Published:** 2026-02-21

**Authors:** Rafał Maciulewski, Angelika Buczyńska-Backiel, Anna Zielińska-Maciulewska, Katarzyna Siewko, Adam Krętowski, Małgorzata Szelachowska

**Affiliations:** 1 Department of Endocrinology, Diabetology and Internal Medicine, Medical University of Bialystok, 15-276 Bialystok, Poland; rafal.maciulewski@umb.edu.pl (R.M.); anna.zielinska-maciulewska@umb.edu.pl (A.Z.-M.); katarzyna.siewko@umb.edu.pl (K.S.); adamkretowski@wp.pl (A.K.); malgorzata.szelachowska@umb.edu.pl (M.S.); 2 Clinical Research Centre, Medical University of Bialystok, 15-276 Bialystok, Poland

**Keywords:** diabetes mellitus, C-peptide, β-cell function, glucagon stimulation test, LADA, insulin resistance, HOMA-IR

## Abstract

Residual β-cell secretory function plays a central role in diabetes pathophysiology; however, long-term comparative data describing β-cell trajectories from diagnosis across diabetes phenotypes remain limited. In this prospective observational study, 393 adults with newly diagnosed diabetes underwent assessment of fasting and glucagon-stimulated C-peptide at diagnosis. The cohort included individuals with type 1 diabetes mellitus (T1DM), encompassing both classical adult-onset autoimmune diabetes and latent autoimmune diabetes in adults (LADA), as well as individuals with type 2 diabetes mellitus (T2DM). A subgroup of 89 participants underwent follow-up visit after a mean of seven years. Glucagon stimulation testing was not repeated at follow-up in patients with T1DM and LADA for clinical and safety reasons; therefore, longitudinal analyses in these groups are based on fasting C-peptide measurements. At diagnosis, fasting and glucagon-stimulated C-peptide concentrations differed markedly between phenotypes (median fasting C-peptide: 0.87 ng/mL in T1DM, 1.53 ng/mL in LADA, and 2.64 ng/mL in T2DM; stimulated C-peptide: 1.35, 1.86, and 4.60 ng/mL, respectively; all *p* < 0.001). During follow-up, patients with T1DM exhibited a pronounced decline in fasting C-peptide (from 0.95 to 0.10 ng/mL), whereas individuals with T2DM showed preserved or increased stimulated responses (from 4.37 to 5.46 ng/mL). Participants with LADA displayed intermediate baseline values and a gradual decline in fasting C-peptide (from 1.41 to 0.31 ng/mL). Higher baseline C-peptide concentrations were associated with more favourable long-term metabolic profiles, including lower insulin resistance and better glycaemic control. These findings demonstrate that the early dynamic assessment of β-cell reserve using the glucagon stimulation test complements fasting C-peptide by revealing biologically meaningful heterogeneity in disease trajectories, thereby refining phenotypic classification and prognostic stratification at the time of diabetes diagnosis.

## 1. Introduction

Diabetes mellitus comprises a heterogeneous group of chronic metabolic disorders characterized by persistent hyperglycaemia resulting from impaired insulin secretion, impaired insulin action, or both [1,2]. It is one of the most prevalent non-communicable diseases worldwide, affecting over 500 million adults and imposing a rapidly increasing global burden with substantial morbidity related to chronic vascular complications [3]. Type 1 diabetes mellitus (T1DM) is primarily driven by immune-mediated destruction of pancreatic β-cells, resulting in near-complete insulin deficiency. In contrast, type 2 diabetes mellitus (T2DM) develops from a complex interplay between insulin resistance and a progressive decline in β-cell secretory capacity, with marked interindividual variability in the relative contribution of these mechanisms over time [4,5].

Latent autoimmune diabetes in adults (LADA), historically regarded as an intermediate entity between T1DM and T2DM, is now recognized as a slowly progressive form of autoimmune T1DM. Although it shares key immunological features with classical T1DM, including the presence of islet autoantibodies (most commonly glutamic acid decarboxylase 65 autoantibodies (anti-GAD65), insulinoma-associated protein 2 autoantibodies (IA-2A), insulin autoantibodies (IAA), zinc transporter 8 autoantibodies (ZnT8A)), LADA is distinguished by a more indolent course, with gradual β-cell loss and delayed insulin dependence [6]. While detection of islet autoantibodies remains the diagnostic standard for autoimmune diabetes, antibody testing is not universally available, incurs additional cost, and may yield equivocal or transient results [4,5,6,7,8,9]. In this context, functional assessment of β-cell reserve using the glucagon stimulation test (GST) may provide complementary and clinically actionable information, particularly in patients with a T2DM-like phenotype in whom autoimmune diabetes might otherwise remain unrecognized [7,8].

Assessment of pancreatic β-cell secretory reserve is central to mechanistic phenotyping of diabetes, as it directly reflects residual endogenous insulin production. C-peptide, secreted equimolarly with insulin during proinsulin processing, enables discrimination between insulin deficiency and insulin resistance independently of exogenous insulin therapy [7,8]. Measurement of fasting and stimulated C-peptide distinguishes basal secretion from dynamic secretory capacity, allowing quantification of functional β-cell reserve at diagnosis [9,10]. The GST provides a standardized and reproducible in vivo measure of acute β-cell function and correlates closely with mixed-meal tolerance testing while requiring substantially less time and procedural complexity [11]. Combined evaluation of fasting and stimulated C-peptide, together with derived indices (area under the curve (AUC), homeostasis model assessment of insulin resistance (HOMA-IR), and homeostasis model assessment of β-cell function (HOMA-%β)), enables integrated characterization of basal secretion, dynamic reserve, and insulin sensitivity.

In T1DM, measurable β-cell activity may persist for years after diagnosis and is associated with lower glycaemic variability, reduced insulin requirements, and a lower risk of acute metabolic complications [12,13]. In T2DM, loss of β-cell secretory capacity is a primary driver of disease progression and a major predictor of secondary failure of glucose-lowering therapy [14,15,16,17,18]. Despite the established clinical relevance of residual insulin secretion, longitudinal data describing phenotype-specific trajectories of β-cell reserve from diagnosis remain scarce. When this study was initiated, LADA was commonly classified as an intermediate form of diabetes; current consensus now recognizes it as a slowly progressive autoimmune variant of T1DM [16,17]. Accordingly, LADA was initially analyzed within the autoimmune diabetes group and subsequently examined as a separate subgroup to capture its distinct metabolic phenotype and intermediate trajectory of β-cell decline.

The aim of this study was to address the lack of longitudinal, phenotype-resolved data on β-cell reserve by comparing fasting and glucagon-stimulated C-peptide at diagnosis and by defining disease-specific trajectories of β-cell secretory capacity over seven years in T1DM and T2DM. By linking these trajectories to concurrent metabolic parameters, we sought to determine how early functional differences translate into divergent long-term metabolic phenotypes and clinical courses.

## 2. Results

### 2.1. Characteristics of Patients with Newly Diagnosed Diabetes

Statistically significant differences were observed between patients with T1DM and T2DM across all analyzed parameters. Patients with newly diagnosed T2DM, compared with those with newly diagnosed T1DM, had significantly higher body mass index (BMI; 30.57 vs. 22.4, *p* < 0.001), waist-to-hip ratio (WHR; 0.99 vs. 0.89, *p* < 0.001), fasting C-peptide concentration (2.64 ng/mL vs. 0.87 ng/mL, *p* < 0.001), C-peptide concentration 6 min after glucagon administration (4.6 ng/mL vs. 1.35 ng/mL, *p* < 0.001), and area under the C-peptide curve (AUC; 22.0 vs. 6.75, *p* < 0.001). T2DM patients also demonstrated higher fasting insulin levels (14.2 mIU/mL vs. 5.7 mIU/mL, *p* < 0.001), total cholesterol (193.5 mg/dL vs. 171 mg/dL, *p* < 0.001), LDL cholesterol (117.0 mg/dL vs. 100.3 mg/dL, *p* < 0.001), triglycerides (134.0 mg/dL vs. 118 mg/dL, *p* < 0.001), HOMA-IR (4.8 vs. 1.9, *p* < 0.001), and HOMA-%β (78.2 vs. 27.1, *p* < 0.001).

Conversely, patients with newly diagnosed T1DM had significantly higher HbA1c levels (10.8% vs. 7.3%, *p* < 0.001), fasting plasma glucose (142.5 mg/dL vs. 130 mg/dL, *p* = 0.011), plasma glucose 6 min after glucagon administration (163 mg/dL vs. 149 mg/dL, *p* = 0.013), and HDL cholesterol levels (48 mg/dL vs. 45 mg/dL, *p* = 0.028) compared with those with newly diagnosed T2DM. These findings demonstrate a profound metabolic and β-cell functional contrast between T1DM and T2DM already at diagnosis, with T2DM characterized by preserved insulin secretion and insulin resistance, and T1DM by severe β-cell failure and poorer glycaemic control. In accordance with current recommendations, patients with LADA were classified within the T1DM group for the main comparative analyses. To account for the distinct clinical and pathophysiological features of adult-onset autoimmune diabetes, LADA patients were additionally analyzed as a separate subgroup in Section 2.3. The characteristics of patients with newly diagnosed diabetes are presented in Table 1.

### 2.2. Correlations Between C-Peptide Concentrations and Clinical Parameters in Patients with T1DM and T2DM at Diagnosis

At diagnosis, patients with T1DM were younger than those with T2DM (median age: 32.5 [24.0–40.5] vs. 55.0 [45.0–62.0] years; *p* < 0.001), as shown in Table 1, reflecting the distinct epidemiological profiles of autoimmune and insulin-resistant diabetes and providing clinical context for the divergent β-cell trajectories observed in subsequent analyses. Both fasting and stimulated C-peptide levels showed strong positive correlations with markers of adiposity, including BMI and WHR.

As expected, fasting and post-glucagon C-peptide concentrations were strongly correlated with each other (R = 0.95, *p* < 0.001), indicating the internal consistency of β-cell secretory assessment. These findings indicate that in newly diagnosed T1DM, residual β-cell function varies in relation to anthropometric and metabolic characteristics at presentation, which may in part reflect the timing of diagnosis during the catabolic phase of disease onset. Moreover, C-peptide concentrations correlated with other markers of endogenous insulin secretion, confirming internal consistency of the analyses.

In patients with newly diagnosed T2DM, both fasting and stimulated C-peptide concentrations were strongly and positively correlated with body weight, BMI and WHR, indicating a close link between β-cell secretory capacity and adiposity. In this group, fasting and stimulated C-peptide also showed significant inverse correlations with HbA1c, demonstrating that lower β-cell reserve is associated with poorer glycaemic control. In addition, C-peptide concentrations correlated positively with fasting insulin and triglyceride levels, consistent with the insulin-resistant metabolic phenotype characteristic of T2DM.

Significant associations between fasting and post-glucagon C-peptide and selected anthropometric and biochemical parameters in patients with newly diagnosed T1DM and T2DM are summarized in Table 2. Collectively, these findings demonstrate that residual β-cell function at diagnosis is closely linked to adiposity and metabolic status, reinforcing the value of C-peptide as a functional integrator of endogenous insulin secretion within the broader metabolic context.

### 2.3. Comparison of Patients with Newly Diagnosed Type 1, Type 2, and LADA Diabetes

At the time of diagnosis, patients with LADA demonstrated significantly lower BMI (24.9 vs. 30.6, *p* < 0.001), WHR (0.92 vs. 0.99, *p* = 0.005), fasting C-peptide concentration (1.53 ng/mL vs. 2.64 ng/mL, *p* < 0.001), and C-peptide concentration 6 min after glucagon administration (1.86 ng/mL vs. 4.6 ng/mL, *p* < 0.001) compared with patients newly diagnosed with T2DM. Similarly, the area under the C-peptide curve (AUC; 10.4 vs. 22.0, *p* < 0.001), fasting insulin concentration (6.1 mIU/mL vs. 14.2 mIU/mL, *p* < 0.001), LDL cholesterol (100.2 mg/dL vs. 117 mg/dL, *p* = 0.023), triglycerides (93.5 mg/dL vs. 134 mg/dL, *p* = 0.002), HOMA-IR (2.4 vs. 4.8, *p* = 0.022), and HOMA-%β (32.2 vs. 78.2, *p* = 0.004) were significantly lower in LADA patients compared with those with T2DM. Conversely, LADA patients exhibited a significantly higher HbA1c percentage (10.6% vs. 7.3%, *p* = 0.004).

No statistically significant differences were found between patients with newly diagnosed LADA and those with newly diagnosed T1DM in the analyzed parameters, which supports the decision to initially analyze patients within broader phenotypic groups rather than separating them a priori, as β-cell function at diagnosis reflects a continuous metabolic spectrum rather than discrete clinical categories. Nevertheless, these results place LADA between classical T1DM and T2DM with respect to metabolic profile and β-cell reserve, supporting its characterization as a slowly progressive form of autoimmune diabetes. The characteristics of patients with newly diagnosed T1DM, T2DM, and LADA are presented in Table 3.

### 2.4. Characteristics of Patients After an Average of Seven Years of Diabetes Duration

After approximately seven years of treatment, patients with T2DM had significantly higher BMI (30.8 vs. 24.4 kg/m^2^, *p* < 0.001), WHR (0.99 vs. 0.85, *p* < 0.001), fasting C-peptide concentration (2.86 vs. 0.10 ng/mL, *p* < 0.001), and triglycerides (119.0 vs. 71.5 mg/dL, *p* < 0.001) compared with patients with T1DM. Conversely, patients with T1DM demonstrated significantly higher HbA1c (8.1% vs. 6.8%, *p* < 0.001) and HDL cholesterol levels (63.5 vs. 44.0 mg/dL, *p* < 0.001). No statistically significant differences were found between the two groups in fasting plasma glucose, total cholesterol, or LDL cholesterol concentrations. Thus, despite long-term treatment, distinct metabolic and β-cell-related differences between T1DM and T2DM persist, reflecting fundamentally different disease trajectories. It should be noted that this analysis includes only the subset of patients who were re-examined after approximately seven years of follow-up. Accordingly, the age ranges reported in this section reflect the age at diagnosis in this follow-up cohort and differ from those of the full baseline population presented in Section 2.3. In accordance with current recommendations, patients with LADA were classified within the T1DM group for the main comparative analyses, as performed in initial evaluation. To account for the distinct clinical and pathophysiological features of adult-onset autoimmune diabetes, LADA patients were additionally analyzed as a separate subgroup in Section 2.8. The characteristics of patients after an average of seven years of diabetes duration are presented in Table 4.

### 2.5. Characteristics of Patients with T1DM, LADA, and T2DM at Diagnosis and Follow-Up After an Average of Seven Years of Disease Duration

Pairwise post hoc analyses revealed significant differences (*p* < 0.05) between T1DM and LADA in age, BMI, WHR, HbA1c, fasting and glucagon-stimulated C-peptide concentrations, C-peptide AUC, insulin levels, total and LDL cholesterol, triglycerides, HOMA-IR, and HOMA-%β. Comparisons between T1DM and T2DM showed significant differences (*p* < 0.05) in all analyzed parameters except HDL cholesterol. In turn, patients with LADA differed significantly from those with T2DM (*p* < 0.05) in age, BMI, WHR, HbA1c, fasting and stimulated C-peptide, C-peptide AUC, insulin, LDL cholesterol, triglycerides, HOMA-IR, and HOMA-%β. The characteristics of patients with T1DM, LADA, and T2DM at diagnosis who were re-evaluated after an average of seven years of disease duration are presented in Table 5. These pairwise differences confirm persistent phenotype-specific metabolic and β-cell patterns over time, with LADA maintaining an intermediate position between T1DM and T2DM. The narrower age ranges observed in this section result from selective participation in long-term follow-up and do not represent age progression over time, but rather the baseline age distribution of individuals available for follow-up. Differences in age and BMI between Table 4 and Table 5 reflect the fact that Table 5 includes only the subset of patients with complete paired baseline and follow-up data, whereas Table 4 summarizes all available patients at follow-up.

### 2.6. Comparison of Selected Parameters in Patients with T1DM at Diagnosis and After an Average of Seven Years of Treatment

After an average of seven years of therapy, patients with T1DM demonstrated significantly higher BMI (24.4 vs. 22.5 kg/m^2^, *p* = 0.024), total cholesterol (194.5 vs. 178.0 mg/dL, *p* = 0.006), and HDL cholesterol (63.5 vs. 47.5 mg/dL, *p* < 0.001) compared to the values at diagnosis. Conversely, these patients exhibited significantly lower HbA1c (8.1% vs. 10.2%, *p* < 0.001), fasting C-peptide (0.10 vs. 0.95 ng/mL, *p* < 0.001), and triglyceride concentrations (71.5 vs. 86.5 mg/dL, *p* = 0.027). No statistically significant differences were observed in WHR, fasting glucose, or LDL cholesterol levels. Changes in the analyzed parameters reflect progressive β-cell failure in T1DM despite metabolic improvement, underscoring the irreversible nature of autoimmune β-cell destruction. These changes between the time of diabetes diagnosis and after an average of seven years of treatment in patients with T1DM are presented in Table 6. Age characteristics of these patient groups were reported in Section 2.1, Section 2.2, Section 2.3, Section 2.4 and Section 2.5 and in Table 1, Table 2, Table 3, Table 4 and Table 5. Section 2.6, Section 2.7 and Section 2.8 present within-subject longitudinal comparisons; therefore, age was not repeated, as each patient serves as their own control in these analyses.

### 2.7. Comparison of Selected Parameters in Patients with T2DM at Diagnosis and After an Average of Seven Years of Treatment

After seven years long-term treatment, patients with T2DM exhibited significantly lower BMI (30.8 vs. 31.6 kg/m^2^, *p* = 0.016), HbA1c (6.8% vs. 7.3%, *p* = 0.024), HOMA-IR (3.6 vs. 4.8, *p* = 0.016), and HOMA-%β (62.0 vs. 78.2, *p* = 0.001), as well as significantly higher stimulated C-peptide at 6 min (5.46 vs. 4.37 ng/mL, *p* = 0.027) and area under the C-peptide curve (AUC; 25.2 vs. 20.2, *p* = 0.029). No significant differences were observed in fasting glucose, total cholesterol, HDL cholesterol, LDL cholesterol, or triglyceride levels. Changes in selected clinical and biochemical parameters between the time of diagnosis and after an average of seven years of treatment in patients with T2DM are shown in Table 7. In T2DM, long-term treatment was associated with improved insulin sensitivity and preserved or enhanced stimulated insulin secretion, indicating sustained β-cell functional capacity.

### 2.8. Prospective Observation of Patients with LADA

When comparing data from nine LADA patients at the time of diagnosis and after an average of seven years of treatment, a statistically significant decrease in fasting C-peptide concentration was observed (0.31 ng/mL vs. 1.41 ng/mL, *p* = 0.011). Other glucagon stimulation test parameters demonstrated trends toward changes without reaching conventional statistical significance, including C-peptide concentration 6 min after glucagon administration (0.74 vs. 1.8 ng/mL, *p* = 0.063) and C-peptide area under the curve (AUC; 3.4 vs. 9.1, *p* = 0.075), suggesting a directional effect that did not attain sufficient statistical power. Thus, the lack of statistical significance for post-glucagon C-peptide and C-peptide AUC likely results from the limited number of LADA patients included in the prospective follow-up. Nevertheless, these findings suggest a gradual but continuous decline in β-cell function in LADA, consistent with a slowly progressive autoimmune process. A prospective observation of patients with LADA is presented in Table 8.

## 3. Discussion

In this prospective study, we demonstrate that glucagon stimulation test (GST) results and baseline biochemical characteristics clearly distinguish T1DM from T2DM, with LADA displaying intermediate features [1,6,16,17]. To our knowledge, this is the first study to describe long-term, stimulation-based trajectories of β-cell reserve from diagnosis across T1DM, LADA, and T2DM. Baseline C-peptide served a dual role: it enabled objective phenotypic classification and acted as a prognostic marker of future metabolic trajectories [7,8,9,10]. Fasting and glucagon-stimulated C-peptide concentrations correlated strongly with anthropometric and metabolic parameters in both T1DM and T2DM, confirming their value as indicators of β-cell secretory reserve [7,8,9,10,11]. These findings indicate that C-peptide is not an isolated biochemical measure but reflects an integrated metabolic phenotype. Higher values identify individuals with preserved β-cell reserve embedded within an insulin-resistant metabolic milieu, whereas lower values mark a state of β-cell failure associated with adverse metabolic profiles. In this way, C-peptide anchors patients along a continuous spectrum between insulin resistance and insulin deficiency, providing both phenotypic and prognostic information. Because C-peptide measurements were obtained after initial metabolic stabilisation, the observed differences are unlikely to reflect transient functional suppression and instead represent true variation in residual β-cell reserve at diagnosis, with minimal influence of acute glucotoxicity, particularly in T1DM and LADA. After approximately seven years, patients with T1DM showed a marked decline in C-peptide, reflecting progressive β-cell loss [12,13]. In this group, the concurrent increase in BMI and decrease in WHR observed during follow-up likely reflect recovery from the catabolic state present at diagnosis and a shift toward more peripheral fat distribution following initiation of insulin therapy.

In contrast, individuals with T2DM exhibited increases in stimulated and AUC C-peptide accompanied by a reduction in HOMA-IR [14,18], indicating an improvement in insulin sensitivity rather than compensatory β-cell hypersecretion. This pattern suggests a metabolically more efficient β-cell response within a less insulin-resistant milieu. These divergent trajectories highlight fundamentally different disease mechanisms operating in autoimmune versus insulin-resistant diabetes. Previous studies have shown that preservation of endogenous insulin secretion confers metabolic and clinical benefits in both T1DM and T2DM [12,13,14,19]; however, the magnitude and clinical expression of these effects vary across phenotypes and disease stages.

In LADA, fasting C-peptide declined significantly over time and remained clearly distinct from T2DM, providing direct longitudinal evidence for an intermediate trajectory of autoimmune β-cell failure and supporting its classification as a slowly progressive form of autoimmune diabetes [6,16,17]. The modest improvement in HbA1c despite measurable residual C-peptide likely reflects the dual pathophysiology of LADA, in which progressive immune-mediated β-cell loss coexists with insulin resistance. In this context, preserved β-cell function may primarily attenuate metabolic deterioration rather than translate into overt glycaemic improvement. Large prospective cohorts support the clinical relevance of residual β-cell function. In the Diabetes Control and Complications Trial (DCCT) and its long-term EDIC follow-up, individuals with higher stimulated C-peptide levels at baseline maintained better glycaemic control, exhibited lower glucose variability, and experienced substantially reduced risks of severe hypoglycaemia and microvascular complications, including retinopathy and nephropathy, irrespective of insulin dose [19,20]. Similarly, data from the Scottish Diabetes Research Network Type 1 Bioresource demonstrated that even low-level residual C-peptide secretion was associated with lower HbA1c, reduced prevalence of retinopathy and microalbuminuria, and fewer hypoglycaemia-related hospital admissions [20]. These observations established the concept that any measurable β-cell activity confers clinically meaningful benefit, underscoring the relevance of our findings. Extending this evidence, studies using continuous or flash glucose monitoring have shown that preserved C-peptide secretion is associated with reduced glucose variability and fewer hypoglycaemic events in adults with T1DM [21,22,23,24,25,26,27,28,29,30,31,32]. Our results are consistent with this pattern and support the physiological relevance of residual endogenous insulin secretion even several years after diagnosis.

With respect to LADA, our findings align with longitudinal studies demonstrating a slower but continuous decline in β-cell function compared with classical T1DM, yet a substantially faster decline than in T2DM [23,24,33,34,35]. Notably, Sørgjerd et al. reported that individuals with low C-peptide levels and high glutamic acid decarboxylase antibody titres progress to insulin dependence significantly earlier than those with preserved β-cell reserve, highlighting the prognostic value of early immuno-metabolic assessment [23]. The decline in fasting and stimulated C-peptide observed in our prospective LADA subgroup mirrors this pattern and underscores the heterogeneity and progressive nature of adult-onset autoimmune diabetes.

The GST proved to be a simple, reproducible, and clinically informative method for assessing β-cell reserve in this cohort. Our results corroborate the findings by Haraguchi et al., who demonstrated a strong correlation between GST-derived C-peptide responses and mixed-meal tolerance testing, supporting reliable estimation of insulin secretory capacity [22]. Compared with more complex protocols, GST remains practical in both clinical and research settings, particularly for longitudinal studies [11,36,37]. Importantly, the present findings gain additional relevance in the era of disease-modifying therapies. Recent trials have demonstrated that immunomodulatory interventions, such as teplizumab, can delay clinical onset and slow β-cell decline in autoimmune diabetes when applied early in the disease course [26]. Our data suggest that early assessment of fasting and stimulated C-peptide could help identify adults with residual β-cell reserve who may derive the greatest benefit from such interventions, particularly within the heterogeneous LADA population.

In T2DM, emerging evidence indicates that early intensive metabolic intervention may preserve β-cell function and alter long-term trajectories [15,38]. The phenotype-specific C-peptide patterns observed in our cohort provide a biological framework for individualized treatment strategies aimed at preserving endogenous insulin secretion.

Although HOMA-derived indices (HOMA-IR and HOMA-%β) provide convenient estimates of insulin resistance and β-cell function in non-insulin-treated individuals, their interpretation requires caution. Previous studies have shown that these surrogate indices lose accuracy in older adults, in individuals with advanced β-cell failure, and when compared with the euglycaemic clamp, the gold standard for assessing insulin sensitivity [25,27]. Accordingly, HOMA-derived indices in the present study were interpreted in conjunction with fasting and stimulated C-peptide measurements, which more directly reflect endogenous insulin secretion. Although HOMA-IR and HOMA-%β are widely used as practical descriptors of metabolic status, they represent indirect estimates rather than physiological measures. Their reliability decreases in insulin-treated states and in advanced β-cell failure, and, in these settings, they may misrepresent true metabolic status. Therefore, in this study, HOMA indices were used only as supportive metabolic descriptors and were always interpreted alongside direct C-peptide measurements, which more accurately capture β-cell secretory capacity.

The heterogeneous trajectories of β-cell reserve observed across diabetes phenotypes may partly reflect underlying genetic susceptibility. Common genetic variants influence intrinsic β-cell resilience, insulin secretory capacity, and vulnerability to metabolic or immune-mediated stress. In T2DM, polymorphisms in genes such as TCF7L2, SLC30A8, and MTNR1B modulate insulin secretion and may contribute to interindividual variability in fasting and stimulated C-peptide levels. In contrast, progression of autoimmune diabetes is strongly shaped by immunogenetic background. High-risk HLA-DR3-DQ2 and HLA-DR4-DQ8 haplotypes, as well as variants in PTPN22, CTLA4, and IL2RA, are associated with more rapid β-cell decline, whereas individuals with LADA often carry lower-risk HLA profiles, consistent with the slower deterioration of C-peptide observed in our cohort [28]. These genetically driven differences underscore the limitations of surrogate indices based on steady-state glucose–insulin relationships and highlight the value of stimulation-based assessment, which captures dynamic secretory capacity and distinguishes intrinsic β-cell failure from secondary metabolic adaptation.

Several limitations should be acknowledged. The limited size of the LADA subgroup, particularly at follow-up, precluded detailed subgroup analyses and may introduce confounding in associations with adiposity; however, this reflects the intrinsic rarity and heterogeneity of adult-onset autoimmune diabetes in clinical practice. Although the mixed-meal tolerance test is considered the reference method for assessing residual β-cell function, the use of GST reflects the historical context and real-world feasibility of long-term follow-up [11]. While GST may be less sensitive for detecting minimal secretion, it reliably captures between-phenotype differences, which was the primary aim of this study. GST was not repeated in all participants at follow-up and was not performed in T1DM, introducing asymmetry in longitudinal assessment. Consequently, dynamic β-cell reserve could be evaluated in T2DM and LADA, but only fasting C-peptide in T1DM, which should be considered when comparing trajectories across phenotypes. In addition, longitudinal changes in C-peptide may partly reflect age-related effects, changes in adiposity, or renal function rather than disease-specific mechanisms alone, as multivariable longitudinal models were not performed. Finally, evolving therapeutic strategies (particularly in T2DM) may have influenced insulin sensitivity and β-cell function. Although insulin-treated patients were excluded, residual confounding by non-insulin therapies remains likely; thus, changes observed in T2DM reflect the combined effects of disease evolution and contemporary treatment rather than natural history alone.

Despite these limitations, the prospective design, long follow-up, and comprehensive biochemical characterization provide robust evidence for the clinical relevance of β-cell reserve at diagnosis. ROC analyses demonstrated that both fasting and glucagon-stimulated C-peptide have strong discriminatory performance for differentiating T1DM from T2DM [7,8,9]. However, baseline C-peptide should be viewed primarily as a continuous biological marker rather than a dichotomous decision variable. Values near cut-offs represent transitional states and should be interpreted in clinical context. In this framework, C-peptide becomes a clinically actionable tool that resolves heterogeneity within broad diabetes categories: in T1DM, it identifies individuals with persistent β-cell activity and distinct trajectories; in T2DM, it discriminates preserved reserve from advanced failure; and in LADA, it captures an intermediate autoimmune phenotype not reliably defined by clinical features alone. Importantly, GST reveals dynamic reserve and uncovers differences not evident under fasting conditions [10,11]. In our cohort, stimulated and AUC C-peptide better captured divergent long-term trajectories, particularly in LADA and T2DM. Thus, GST extends C-peptide from a static biomarker to a functional probe of β-cell resilience, with implications for early risk stratification and personalized therapy.

## 4. Material and Methods

### 4.1. Study Design and Study Population

This prospective clinical case–control study was carried out over the period from 2010 to 2022. The screened study cohort consisted of 398 individuals with newly diagnosed diabetes who voluntarily enrolled at the Department of Endocrinology, Diabetology and Internal Medicine of the University Clinical Hospital in Bialystok. During the follow-up phase, conducted approximately seven years after the initial diagnosis, 96 participants underwent follow-up visit.

Before the enrollment, all subjects received comprehensive information about the study and provided written informed consent following consultation with the investigator. Prior to inclusion, a comprehensive medical interview was conducted for each participant, covering previous and chronic medical conditions as well as lifestyle factors such as tobacco use and alcohol intake. Individuals with secondary forms of diabetes (including those associated with glucocorticoid treatment, Cushing’s syndrome, or acute or chronic pancreatitis), liver cirrhosis, end-stage renal disease, malignancies, severe heart failure classified as New York Heart Association (NYHA) class III–IV, or active inflammatory disorders were excluded from the analysis. Additionally, five participants were excluded due to diagnoses other than type 1 or type 2 diabetes mellitus, including one case of type B insulin resistance, one case of pancreatic cancer, and three cases of maturity-onset diabetes of the young (MODY). Diabetes was diagnosed in accordance with the World Health Organization (WHO) diagnostic criteria established in 1999. Latent autoimmune diabetes in adults (LADA) was identified using the definition proposed by Tuomi et al. [16]. Information on treatment was obtained from patients and verified using medical records. Laboratory parameters were not self-reported; all biochemical measurements were performed in a certified laboratory according to standardized procedures at both baseline and follow-up. Autoimmune diabetes was defined by the presence of at least one circulating islet autoantibody (anti-GAD, IA-2A, or IAA). LADA was operationally identified in individuals with adult-onset diabetes who were positive for islet autoantibodies and did not require insulin therapy for at least 6 months after diagnosis. Patients with immediate insulin dependence and positive autoantibodies were classified as classical adult-onset T1DM.

Historically, LADA was considered a distinct form of diabetes, separate from both T1DM and T2DM. In contemporary classification, however, it is regarded as part of the autoimmune diabetes spectrum and is now conceptualized as a slowly progressive form of T1DM. For the present analysis, individuals fulfilling contemporary criteria for LADA (adult-onset diabetes, positivity for islet autoantibodies, and delayed insulin dependence) were retrospectively identified. In accordance with current classification, these patients were initially included within the T1DM group as part of the autoimmune diabetes spectrum and were subsequently analyzed as a separate subgroup to capture their distinct metabolic phenotype and intermediate trajectory of β-cell decline.

Based on study inclusion criteria and current recommendation [1,17], 393 enrolled participants were stratified into two groups: individuals with type 1 diabetes mellitus (T1DM; *n* = 104), including 23 patients diagnosed with LADA, and those with type 2 diabetes mellitus (T2DM; *n* = 289). During the follow-up phase, conducted approximately seven years after the initial diagnosis, 89 patients completed the follow-up assessment (T1DM; *n* = 30, including 9 with LADA, and T2DM; *n* = 59).

### 4.2. Study Procedures and Measurements

In participants presenting with markedly elevated blood glucose levels, fasting blood samples were obtained after prior metabolic stabilization, which included normalization of glycemia with insulin therapy and correction of fluid and electrolyte disturbances. In contrast, among patients without severe hyperglycemia who did not require hospitalization, blood sampling and the GST were performed before the initiation of any antihyperglycemic treatment.

Fasting blood samples were used for the assessment of glucose, C-peptide, total cholesterol, low-density lipoprotein cholesterol (LDL-C), high-density lipoprotein cholesterol (HDL-C), triglycerides (TG), C-reactive protein (CRP), creatinine, glycated hemoglobin (HbA1c), aspartate aminotransferase (AST), and alanine aminotransferase (ALT). Biochemical parameters (glucose, total cholesterol, LDL-C, HDL-C, TG, CRP, creatinine, AST, and ALT) were measured colorimetrically using the Roche C111 analyzer (Roche Diagnostics GmbH, Mannheim, Germany), while hormone concentrations were determined by electrochemiluminescence immunoassay (ECLIA) on the Roche E411 analyzer (Roche Diagnostics GmbH, Mannheim, Germany). Glycated hemoglobin (HbA1c) was assessed by high-performance liquid chromatography (HPLC) using the D-10 analyzer (Bio-Rad Laboratories, Hercules, CA, USA). In addition, circulating autoantibodies (anti-GAD, IA-2A, IAA, and ZnT8A) were determined using enzyme-linked immunosorbent assays (ELISA) with commercially available kits: anti-GAD ELISA (cat. no. EA 1022-9601 G; Euroimmun, Lübeck, Germany), anti-IA2 ELISA (cat. no. EA 1023-9601 G; Euroimmun), anti-GAD/IA2 ELISA (cat. no. EA 1022-9601-1 G; Euroimmun), anti-ZnT8 ELISA (cat. no. EA 1027-9601 G; Euroimmun, Germany), and Medizym® anti-IAA ELISA (cat. no. 3806; Medipan GmbH, Dahlewitz, Germany). Subsequently, a glucagon stimulation test (GST) was conducted by intravenous administration of 1 mg of glucagon. Serum C-peptide and glucose concentrations were measured in the fasting state and again 6 min after glucagon injection. Glucagon concentrations were measured using a radioimmunoassay (RIA) kit (Glucagon RIA, cat. no. GL-32K; Merck Millipore, Darmstadt, Germany), following the manufacturer’s instructions.

The GST was selected as a standardized and practical approach for evaluating stimulated C-peptide secretion. At the time the study was initiated, this test was widely used in both routine clinical practice and longitudinal research due to its short duration and high reproducibility [36,37,38]. Although the mixed-meal tolerance test (MMTT) is currently regarded as the reference method, previous studies have demonstrated a strong correlation between GST- and MMTT-derived C-peptide responses, supporting the reliability of GST in assessing clinically meaningful β-cell secretory capacity.

### 4.3. β-Cell Function Assessment: GST

The glucagon stimulation test was performed according to the consensus recommendations for the assessment of β-cell function, as previously described by Greenbaum et al. [36]. The GST consisted of intravenous administration of 1 mg of glucagon. Blood samples were collected in the fasting state at baseline (0 min) and again 6 min after glucagon injection for the determination of serum C-peptide and glucose levels. The area under the curve (AUC) for C-peptide during the GST was calculated using the formula: AUC = 3 × (C-peptide_0_′ + C-peptide_6_′), where C-peptide_0_′ denotes the fasting concentration measured before glucagon administration and C-peptide_6_′ represents the concentration measured 6 min after glucagon injection.

### 4.4. Follow-Up Visits

After a mean follow-up of approximately seven years, 96 participants agreed to participate in the second stage of the study, of whom 89 completed the follow-up assessment. Seven individuals were excluded at this stage: three who achieved normoglycemia after bariatric surgery and four who were referred for genetic testing to verify diabetes classification. Of the original cohort of 393 participants, five had died, 38 were lost to follow-up, and 259 declined follow-ups but provided information on their current treatment. In addition, 14 participants were retrospectively reclassified as LADA based on positive islet autoantibodies and the initiation of insulin therapy at least six months after diagnosis. Patients with T2DM were managed with lifestyle modification and oral glucose-lowering agents. To reduce heterogeneity associated with advanced β-cell dysfunction, individuals with T2DM who required insulin therapy during follow-up were also excluded from further analyses.

The follow-up cohort included 30 patients with T1DM, including 9 with LADA, and 59 patients with T2DM. During the follow-up period, diabetes treatment was conducted according to standard clinical practice and current guidelines. All patients with T1DM received insulin therapy, and four met criteria for genetic testing.

At follow-up, fasting blood samples were collected to measure glucose, C-peptide, total cholesterol, LDL-C, HDL-C, TG, CRP, creatinine, HbA1c, AST, ALT, thyroid-stimulating hormone (TSH), free thyroxine (fT4), and free triiodothyronine (fT3). The urinary albumin-to-creatinine ratio (ACR) was determined from a spot urine sample. In T1DM patients, GST was not repeated. Anthropometric measurements were reassessed using the same methods described above.

### 4.5. Anthropometric Measurements

Anthropometric assessment comprised measurements of body weight obtained with an electronic scale, body height, waist and hip circumferences, and the waist-to-hip ratio (WHR). Body mass index (BMI) was calculated according to the standard formula.

### 4.6. Homeostatic Model Assesment

Homeostatic model assessment indices were calculated in patients not treated with insulin according to the formulas [18]:

HOMA-IR = glucose (mg/dL) × insulin (mIU/mL)/405

HOMA-%β = [360 × insulin (mIU/mL)]/[glucose (mg/dL) − 63].

### 4.7. Sample Size Considerations

The study was conducted as a prospective, exploratory observational analysis with consecutive enrollment of adults with newly diagnosed diabetes. Owing to its exploratory nature, no formal a priori sample size or power calculation was undertaken. Instead, the number of participants was determined pragmatically, based on the availability of eligible patients and the feasibility of long-term follow-up, rather than on predefined hypotheses or expected effect sizes.

Marked differences in fasting and glucagon-stimulated C-peptide concentrations between T1DM and T2DM at diagnosis have been consistently reported in the literature, with large effect sizes [19,20,21,22]. In the present cohort, the between-group differences in both fasting and stimulated C-peptide levels substantially exceeded within-group variability, enabling reliable discrimination between diabetes phenotypes even with moderate sample sizes. The baseline sample size was sufficient to provide robust estimates of central tendency and variability for key metabolic parameters and to allow valid nonparametric comparisons between groups. Although the number of participants available for follow-up was reduced due to the extended observation period, the follow-up cohort remained adequate for descriptive longitudinal analyses and for evaluating within-group changes over time. The adequacy of the sample size was further supported by post hoc evaluation of observed effect sizes, confirming sufficient power to detect clinically meaningful differences in fasting and glucagon-stimulated C-peptide concentrations between diabetes phenotypes at baseline.

### 4.8. Statistical Analysis

Statistical analyses were performed using STATISTICA software (version 13.3; StatSoft Inc., Tulsa, OK, USA). A two-sided *p* value < 0.05 was considered statistically significant. Data distribution was assessed using the Shapiro–Wilk test. As most variables were not normally distributed, nonparametric statistical methods were applied, and results are presented as medians with interquartile ranges (Q1–Q3). Comparisons between two groups were performed using the Mann–Whitney U test, while comparisons among three groups were conducted with the Kruskal–Wallis test followed by post hoc analyses. Associations between variables were evaluated using Spearman’s rank correlation coefficient. Receiver operating characteristic (ROC) curve analysis was used to assess the ability of fasting and stimulated C-peptide concentrations to discriminate between T1DM and T2DM. The AUC was calculated, and optimal cut-off values were identified using the Youden index to maximize sensitivity and specificity.

## 5. Conclusions

This study shows that β-cell secretory capacity at diagnosis is a primary driver of long-term metabolic trajectory. Higher fasting and glucagon-stimulated C-peptide identified patients with lower insulin resistance and more favorable anthropometric and biochemical profiles. GST provided a direct, reproducible measure of functional β-cell reserve and closely tracked clinically relevant metabolic status. Over seven years, β-cell function declined rapidly in T1DM, was preserved or increased in T2DM, and followed an intermediate course in LADA, defining distinct phenotype-specific trajectories. These data demonstrate that early application of GST, by revealing stimulated C-peptide responses, captures biologically meaningful heterogeneity in disease trajectories. In clinical practice, assessment of β-cell reserves at diagnosis can refine phenotypic classification, identify individuals at risk of rapid β-cell failure, and support the earlier implementation of phenotype-adapted therapeutic strategies.

## Figures and Tables

**Table 1:** Baseline characteristics of patients with newly diagnosed Type 1 and Type 2 diabetes.

Parameter	Type 1 Diabetes (*n* = 104)	Type 2 Diabetes (*n* = 289)	*p* Value
Age (years)	32.5 (24.0–40.5)	55.0 (45.0–62.0)	<0.001
BMI (kg/m^2^)	22.4 (20.6–24.8)	30.57 (27.1–34.1)	<0.001
WHR	0.89 (0.83–0.94)	0.99 (0.93–1.03)	<0.001
HbA1c (%)	10.8 (9.1–12.7)	7.3 (6.0–10.4)	<0.001
C-peptide 0′ (ng/mL)	0.87 (0.51–1.46)	2.64 (1.8–3.56)	<0.001
C-peptide 6′ (ng/mL)	1.35 (0.84–2.12)	4.6 (3.1–6.6)	<0.001
C-peptide AUC	6.75 (4.1–10.9)	22.0 (15.3–30.0)	<0.001
Insulin (mIU/mL)	5.7 (3.9–9.0)	14.2 (9.2–20.5)	<0.001
Glucose 0′ (mg/dL)	142.5 (123–178)	130.0 (112.0–160.0)	0.011
Glucose 6′ (mg/dL)	163.0 (138–197)	149.0 (130.5–176.0)	0.013
Total cholesterol (mg/dL)	171.0 (145.0–195.0)	193.5 (165.0–229.0)	<0.001
HDL cholesterol (mg/dL)	48.0 (39.0–59.0)	45.0 (35.0–53.5)	0.028
LDL cholesterol (mg/dL)	100.3 (75.5–121.5)	117.0 (90.2–146.0)	<0.001
Triglycerides (mg/dL)	118.6 (69.0–126.0)	134.0 (102.0–191.0)	<0.001
HOMA-IR	1.9 (1.4–3.3)	4.8 (3.2–7.4)	<0.001
HOMA-%β	27.1 (16.4–42.3)	78.2 (41.4–131.3)	<0.001

Data are presented as median (interquartile range). Statistical significance was determined using the Mann–Whitney U test. Abbreviations: BMI—body mass index; WHR—waist-to-hip ratio; HbA1c—glycated hemoglobin; AUC—area under the curve; HOMA-IR—homeostatic model assessment for insulin resistance; HOMA-%β—homeostatic model assessment of β-cell function.

**Table 2:** Correlations between fasting and glucagon-stimulated C-peptide concentrations and selected clinical parameters in patients with newly diagnosed T1DM and T2DM.

Parameter	Fasting C-Peptide		C-Peptide 6 Min After Glucagon	
	T1DM			
	R	*p*	R	*p*
Body weight	0.26	0.008	0.27	0.007
BMI	0.38	<0.001	0.36	<0.001
Hip circumference	0.25	0.015	0.23	0.020
Waist circumference	0.35	<0.001	0.35	<0.001
WHR	0.36	0.001	0.39	<0.001
C-peptide 6′	0.95	<0.001	—	—
C-peptide AUC	0.98	<0.001	0.99	<0.001
Insulin	0.41	<0.001	0.39	<0.001
	T2DM			
	R	*p*	R	*p*
Body weight	0.27	<0.001	0.16	0.008
BMI	0.38	<0.001	0.29	<0.001
Hip circumference	0.30	<0.001	0.23	<0.001
Waist circumference	0.30	<0.001	0.22	<0.001
HbA1c	–0.16	0.008	–0.20	<0.001
C-peptide 6′	0.89	<0.001	—	—
C-peptide AUC	0.95	<0.001	0.99	<0.001
Insulin	0.58	<0.001	0.51	<0.001
Triglycerides	0.22	<0.001	0.17	0.004

Data represent Spearman’s rank correlation coefficients (R). All correlations shown were statistically significant (*p* < 0.05). Abbreviations: BMI—body mass index; WHR—waist-to-hip ratio; HbA1c—glycated hemoglobin; AUC—area under the curve.

**Table 3:** Baseline characteristics of patients with newly diagnosed T1DM, LADA, and T2DM.

Parameter	T1DM (*n* = 81)	LADA (*n* = 23)	T2DM (*n* = 289)	*p* Value
Age (years)	28.0 (23.0–35.0)	45.0 (37.0–62.0)	55.0 (45.0–62.0)	<0.001
BMI (kg/m^2^)	21.9 (20.3–24.2)	24.9 (23.0–28.0)	30.6 (27.1–34.1)	<0.001
WHR	0.88 (0.82–0.92)	0.92 (0.85–0.97)	0.99 (0.93–1.03)	<0.001
HbA1c (%)	10.8 (9.4–12.8)	10.6 (7.5–11.8)	7.3 (6.0–10.4)	<0.001
C-peptide 0′ (ng/mL)	0.81 (0.48–1.23)	1.53 (0.78–1.89)	2.64 (1.8–3.56)	<0.001
C-peptide 6′ (ng/mL)	1.20 (0.80–2.00)	1.86 (1.20–3.43)	4.60 (3.10–6.60)	<0.001
C-peptide AUC	5.90 (3.60–9.60)	10.4 (5.9–15.8)	22.0 (15.3–30.0)	<0.001
Insulin (mIU/mL)	4.8 (3.7–8.0)	6.1 (5.5–10.9)	14.2 (9.2–20.5)	<0.001
Glucose 0′ (mg/dL)	141.0 (120.0–176.0)	147.0 (127.0–181.0)	130.0 (112.0–160.0)	0.033
Glucose 6′ (mg/dL)	162.0 (137.5–195.5)	163.0 (140.0–204.0)	149.0 (130.5–176.0)	0.045
Total cholesterol (mg/dL)	171.0 (141.5–195.5)	170.0 (155.0–195.0)	193.5 (165.0–229.0)	<0.001
HDL cholesterol (mg/dL)	47.0 (39.0–58.0)	49.5 (44.0–67.0)	45.0 (35.0–53.5)	0.047
LDL cholesterol (mg/dL)	101.0 (76.0–125.0)	100.2 (73.0–113.0)	117.0 (90.2–146.0)	<0.001
Triglycerides (mg/dL)	88.5 (69.0–126.0)	93.5 (59.0–131.0)	134.0 (102.0–191.0)	<0.001
HOMA-IR	1.8 (1.3–3.2)	2.4 (1.8–7.3)	4.8 (3.2–7.4)	<0.001
HOMA-%β	26.9 (15.9–40.4)	32.2 (20.9–50.7)	78.2 (41.4–131.3)	<0.001

Data are presented as median (interquartile range). Statistical significance was assessed using the Kruskal–Wallis test with post hoc analysis. Abbreviations: BMI—body mass index; WHR—waist-to-hip ratio; HbA1c—glycated hemoglobin; AUC—area under the curve; HOMA-IR—homeostatic model assessment for insulin resistance; HOMA-%β—homeostatic model assessment of β-cell function.

**Table 4:** Clinical and biochemical characteristics of patients with T1DM and T2DM after a mean of seven years of disease duration.

Parameter	T1DM (*n* = 30)	T2DM (*n* = 59)	*p* Value
Age (years)	41.5 (33.0–51.0)	61.0 (52.0–65.0)	<0.001
BMI (kg/m^2^)	24.4 (22.6–25.2)	30.8 (28.5–34.8)	<0.001
WHR	0.85 (0.82–0.89)	0.99 (0.95–1.05)	<0.001
HbA1c (%)	8.11 (7.0–9.3)	6.8 (6.1–7.4)	<0.001
C-peptide 0′ (ng/mL)	0.10 (0.03–0.36)	2.86 (1.96–3.63)	<0.001
C-peptide 6′ (ng/mL)	not assessed	5.46 (4.0–6.75)	–
C-peptide AUC	not assessed	25.2 (19.2–30.2)	–
Insulin (mIU/mL)	not assessed	10.7 (7.5–16.4)	–
Glucose 0′ (mg/dL)	157.5 (118.0–209.0)	131.0 (109.0–163.0)	0.211
Glucose 6′ (mg/dL)	not assessed	149.0 (130.5–176.0)	–
Total cholesterol (mg/dL)	194.5 (169.0–230.0)	187.0 (149.0–217.0)	0.325
HDL cholesterol (mg/dL)	63.5 (57.0–80.0)	44.0 (38.0–55.0)	<0.001
LDL cholesterol (mg/dL)	113.5 (80.0–149.0)	112.0 (91.0–146.0)	0.801
Triglycerides (mg/dL)	71.5 (56.0–81.0)	119.0 (101.0–161.0)	<0.001

Data are presented as median (interquartile range). Statistical significance was assessed using the Mann–Whitney U test. Abbreviations: BMI—body mass index; WHR—waist-to-hip ratio; HbA1c—glycated hemoglobin; AUC—area under the curve.

**Table 5:** Clinical and biochemical characteristics at diagnosis and after a mean of seven years of follow-up in patients with T1DM, LADA, and T2DM.

Parameter	T1DM (*n* = 21)	LADA (*n* = 9)	T2DM (*n* = 59)	*p* Value
Age (years)	31.0 (24.0–35.0)	43.0 (35.0–45.0)	54.0 (46.0–59.0)	<0.001
BMI (kg/m^2^)	21.9 (19.9–24.4)	24.4 (23.1–25.8)	31.6 (29.0–35.5)	<0.001
WHR	0.87 (0.82–0.89)	0.90 (0.83–0.99)	0.98 (0.94–1.02)	<0.001
HbA1c (%)	10.3 (9.4–14.1)	9.8 (6.8–11.3)	7.3 (6.1–9.2)	<0.001
C-peptide 0′ (ng/mL)	0.8 (0.6–1.0)	1.41 (1.0–1.7)	2.4 (1.7–3.3)	<0.001
C-peptide 6′ (ng/mL)	1.2 (0.9–1.5)	1.8 (1.4–2.1)	4.4 (2.9–6.3)	<0.001
C-peptide AUC	6.5 (4.7–7.1)	9.1 (7.2–11.7)	20.2 (14.3–27.7)	<0.001
Insulin (mIU/mL)	4.8 (3.4–6.7)	5.9 (4.9–7.7)	15.7 (10.4–22.9)	<0.001
Glucose 0′ (mg/dL)	127.0 (113.0–160.0)	165.0 (127.0–184.0)	131.5 (116.0–146.0)	0.207
Glucose 6′ (mg/dL)	152.0 (126.0–182.0)	179.0 (139.0–204.0)	149.5 (131.0–166.0)	0.203
Total cholesterol (mg/dL)	179.0 (164.0–197.0)	162.0 (128.0–187.0)	191.0 (167.0–231.0)	0.045
HDL cholesterol (mg/dL)	46.0 (41.0–67.0)	48.0 (46.0–69.0)	47.0 (37.0–55.0)	0.227
LDL cholesterol (mg/dL)	113.7 (88.7–132.4)	81.4 (53.0–100.5)	116.6 (92.1–157.6)	0.006
Triglycerides (mg/dL)	85.0 (65.0–121.0)	88.0 (60.0–113.0)	131.0 (112.0–174.0)	<0.001
HOMA-IR	1.6 (1.1–2.8)	2.0 (1.8–3.2)	5.3 (3.6–7.4)	<0.001
HOMA-%β	27.1 (16.7–42.3)	25.9 (20.9–51.1)	93.1 (46.5–120.9)	<0.001

Data are presented as median (interquartile range). Statistical significance (*p* < 0.05) was assessed using the Kruskal–Wallis test with post hoc analysis. Abbreviations: BMI—body mass index; WHR—waist-to-hip ratio; HbA1c—glycated hemoglobin; AUC—area under the curve; HOMA-IR—homeostatic model assessment for insulin resistance; HOMA-%β—homeostatic model assessment of β-cell function.

**Table 6:** Comparison of clinical and biochemical parameters in patients with T1DM at diagnosis and after a mean of seven years of follow-up.

Parameter	T1DM at Diagnosis (*n* = 30)	T1DM After Treatment (*n* = 30)	*p* Value
BMI (kg/m^2^)	22.5 (21.1–24.5)	24.4 (22.6–25.2)	0.024
WHR	0.87 (0.83–0.91)	0.85 (0.82–0.89)	0.301
HbA1c (%)	10.2 (8.4–13.1)	8.11 (7.0–9.3)	<0.001
C-peptide 0′ (ng/mL)	0.95 (0.58–1.26)	0.10 (0.03–0.36)	<0.001
Glucose 0′ (mg/dL)	131.0 (113.0–170.0)	157.5 (118.0–209.0)	0.329
Total cholesterol (mg/dL)	178.0 (154.0–195.0)	194.5 (169.0–230.0)	0.006
HDL cholesterol (mg/dL)	47.5 (42.0–68.0)	63.5 (57.0–80.0)	<0.001
LDL cholesterol (mg/dL)	101.3 (77.1–124.0)	113.5 (80.0–149.0)	0.183
Triglycerides (mg/dL)	86.5 (64.0–121.0)	71.5 (56.0–81.0)	0.027

Data are presented as median (interquartile range). Statistical significance was assessed using the Wilcoxon signed-rank test for paired samples. Abbreviations: BMI—body mass index; WHR—waist-to-hip ratio; HbA1c—glycated hemoglobin.

**Table 7:** Comparison of clinical and biochemical parameters in patients with T2DM at diagnosis and after a mean of seven years of follow-up.

Parameter	T2DM at Diagnosis (*n* = 59)	T2DM After Treatment (*n* = 59)	*p* Value
BMI (kg/m^2^)	31.6 (29.0–35.5)	30.8 (28.5–34.8)	0.016
WHR	0.98 (0.94–1.02)	0.99 (0.95–1.05)	0.615
HbA1c (%)	7.3 (6.1–9.2)	6.8 (6.1–7.4)	0.024
C-peptide 0′ (ng/mL)	2.42 (1.68–3.33)	2.86 (1.96–3.63)	0.299
C-peptide 6′ (ng/mL)	4.37 (2.91–6.31)	5.46 (4.0–6.75)	0.027
C-peptide AUC	20.2 (14.3–27.7)	25.2 (19.2–30.2)	0.029
Glucose 0′ (mg/dL)	131.5 (116.0–146.0)	131.0 (109.0–163.0)	0.205
Glucose 6′ (mg/dL)	149.5 (131.0–166.0)	149.0 (130.5–176.0)	0.189
Total cholesterol (mg/dL)	191.0 (167.0–231.0)	187.0 (149.0–217.0)	0.145
HDL cholesterol (mg/dL)	47.0 (37.0–55.0)	44.0 (38.0–55.0)	0.370
LDL cholesterol (mg/dL)	116.6 (92.1–157.6)	112.0 (91.0–146.0)	0.763
Triglycerides (mg/dL)	131.0 (112.0–174.0)	119.0 (101.0–161.0)	0.310
HOMA-IR	4.8 (3.2–7.4)	3.6 (2.3–5.5)	0.016
HOMA-%β	78.2 (41.4–131.3)	62.0 (33.2–89.4)	0.001

Data are presented as median (interquartile range). Statistical significance was assessed using the Wilcoxon signed-rank test for paired samples. Abbreviations: BMI—body mass index; WHR—waist-to-hip ratio; HbA1c—glycated hemoglobin; AUC—area under the curve; HOMA-IR—homeostatic model assessment for insulin resistance; HOMA-%β—homeostatic model assessment of β-cell function.

**Table 8:** Comparison of clinical and biochemical parameters in patients with LADA at diagnosis and after a mean of seven years of follow-up.

Parameter	LADA at Diagnosis (*n* = 9)	LADA After Treatment (*n* = 9)	*p* Value
BMI (kg/m^2^)	24.4 (23.1–25.8)	23.5 (22.1–25.1)	0.310
WHR	0.90 (0.83–0.99)	0.83 (0.82–0.90)	0.345
HbA1c (%)	9.8 (6.8–11.3)	8.9 (7.8–9.3)	0.374
C-peptide 0′ (ng/mL)	1.41 (1.0–1.7)	0.31 (0.04–0.38)	0.011
C-peptide 6′ (ng/mL)	1.8 (1.4–2.1)	0.74 (0.05–1.04)	0.063
C-peptide AUC	9.1 (7.2–11.7)	3.4 (1.8–4.9)	0.075
Glucose 0′ (mg/dL)	165.0 (127.0–184.0)	158.0 (144.0–194.0)	0.515
Glucose 6′ (mg/dL)	179.0 (139.0–204.0)	180.0 (174.0–238.0)	0.353
Total cholesterol (mg/dL)	162.0 (128.0–187.0)	178.0 (158.0–223.0)	0.066
HDL cholesterol (mg/dL)	48.0 (46.0–69.0)	64.0 (54.0–74.0)	0.173
LDL cholesterol (mg/dL)	81.4 (53.0–100.5)	132.0 (78.0–139.0)	0.093
Triglycerides (mg/dL)	88.0 (60.0–113.0)	70.0 (57.0–81.0)	0.110

Data are presented as median (interquartile range). Statistical significance was assessed using the Wilcoxon signed-rank test for paired samples. Abbreviations: BMI—body mass index; WHR—waist-to-hip ratio; HbA1c—glycated hemoglobin; AUC—area under the curve.

## Data Availability

The datasets generated and analyzed during the current study are available from the corresponding author upon reasonable request.
